# Case report: Gitelman syndrome with diabetes: Confirmed by both hydrochlorothiazide test and genetic testing

**DOI:** 10.1097/MD.0000000000033959

**Published:** 2023-06-16

**Authors:** Luyang Yang, Jinmeng Fan, Yunfeng Liu, Yi Ren, Zekun Liu, Hairui Fu, Hao Qi, Jing Yang

**Affiliations:** a Department of Endocrinology, First Hospital of Shanxi Medical University, Taiyuan, Shanxi Province, China; b Shanxi Medical University, Taiyuan, Shanxi Province, China; c Department of Orthopedics, Affiliated Fenyang Hospital of Shanxi Medical University, Fenyang, Shanxi Province, China; d Department of Orthopedics, Affiliated Bethune Hospital of Shanxi Medical University, Taiyuan, Shanxi Province, China.

**Keywords:** Gitelman syndrome, hydrochlorothiazide test, hypokalemia, type 2 diabetes

## Abstract

Gitelman syndrome (GS) is an autosomal recessive tubulopathy caused by mutations of the SLC12A3 gene. It is characterized by hypokalemic metabolic alkalosis, hypomagnesemia and hypocalciuria. Hypokalemia, hypomagnesemia, and increased renin-angiotensin-aldosterone system (RAAS) activity can cause glucose metabolism dysfunction. The diagnosis of GS includes clinical diagnosis, genetic diagnosis and functional diagnosis. The gene diagnosis is the golden criterion while as functional diagnosis is of great value in differential diagnosis. The hydrochlorothiazide (HCT) test is helpful to distinguish GS from batter syndrome, but few cases have been reported to have HCT testing.

**Patient concerns:**

A 51-year-old Chinese woman presented to emergency department because of intermittent fatigue for more than 10 years.

**Diagnoses:**

Laboratory test results showed hypokalemia, hypomagnesemia, hypocalciuria and metabolic alkalosis. The HCT test showed no response. Using next-generation and Sanger sequencing, we identified 2 heterozygous missense variants (c.533C > T:p.S178L and c.2582G > A:p.R861H) in the SLC12A3 gene. In addition, the patient was diagnosed with type 2 diabetes mellitus 7 years ago. Based on these findings, the patient was diagnosed with GS with type 2 diabetic mellitus (T2DM).

**Interventions:**

She was given potassium and magnesium supplements, and dapagliflozin was used to control her blood glucose.

**Outcomes:**

After treatments, her fatigue symptoms were reduced, blood potassium and magnesium levels were increased, and blood glucose levels were well controlled.

**Lessons:**

When GS is considered in patients with unexplained hypokalemia, the HCT test can be used for differential diagnosis, and genetic testing can be continued to confirm the diagnosis when conditions are available. GS patients often have abnormal glucose metabolism, which is mainly caused by hypokalemia, hypomagnesemia, and secondary activation of RAAS. When a patient is diagnosed with GS and type 2 diabetes, sodium-glucose cotransporter 2 inhibitors (SGLT2i) can be used to control the blood glucose level and assist in raising blood magnesium.

## 1. Introduction

Gitelman syndrome (GS) is a rare autosomal recessive renal tubular disease that generally occurs in adolescence or adulthood, mainly due to mutations in the SLC12A3 gene, which leads to abnormalities in the thiazide sensitive sodium chloride cotransporter (NCCT) in the distal convoluted tubules. The main clinical manifestations are hypokalemia, hypomagnesium, hypocalcemia metabolic alkalosis, and enhanced renin-angiotensin-aldos-terone system (RAAS) activity.^[1]^ The diagnosis of GS mostly relies on genetic testing. However, various factors have limited the use of genetic testing, such as the high cost, ethical issues, and the time-consuming nature of the test. The hydrochlorothiazide (HCT) test can help determine the site of renal tubular injury and evaluate the function of NCCT, which can be used as an auxiliary clinical diagnostic tool for GS. Hypokalemia, hypomagnesium, and enhanced RAAS activity in GS patients could lead to abnormal glucose metabolism.^[2–7]^ In this study, we identified 2 previously reported missense mutation c.533C > T:p.S178L and c.2582G > A:p.R861H in a GS patient coexisting with type 2 diabetic mellitus (T2DM). In addition, we explored the common mutations in GS patients, the advantages of HCT testing, the mechanisms of GS-induced hyperglycemia, and possible glucose-lowering regimens.

## 2. Case report

A 51-year-old Chinese woman suffered from dizziness, fatigue, and tetany after catching a cold more than 10 years ago, which was spontaneously relieved half an hour later. She was treated at a local clinic and only given potassium and calcium supplementation without clarifying the cause, and the above symptoms did not recur. In the past 3 months, the patient developed weakness in the limbs, difficulty in lifting legs, dizziness, sweating, cough, yellow phlegm, no vomiting or diarrhea, and she denied using diuretics, licorice preparations, cottonseed oil, and barium chloride. After that, the patient was admitted to the emergency department of our hospital for a serum potassium test of 2.49 mmol/L and a serum calcium test of 1.93 mmol/L. Her fatigue symptoms were relieved after being given symptomatic treatment such as antibiotics, potassium supplements, and fluid replenishment. She was then admitted to the Endocrinology Department of our hospital with the cause of hypokalemia pending investigation.

She was diagnosed with type 2 diabetes mellitus 7 years ago. She had stable blood sugar control with oral metformin and repaglinide daily. Chronic bronchitis was diagnosed 1 year ago and was not treated with medication. She was diagnosed with herpes zoster 2 months ago and has no pain and no medication. In 2015, she underwent cholecystectomy. Any other medical history is denied. She started smoking at the age of 44 with about 10 cigarettes daily. There were no similar cases in her family.

On admission, her weight was 70.0 kg and her height was 166.0 cm. Her body temperature was 36.3°C, her pulse rate was 77 beats per minute, her respiration rate was 19 breaths per minute, and her blood pressure was 117/77 mm Hg. Her muscle strength and muscle tone in her extremities were normal, and symptoms of thyroid and other systems were not obvious.

**Figure 1.**
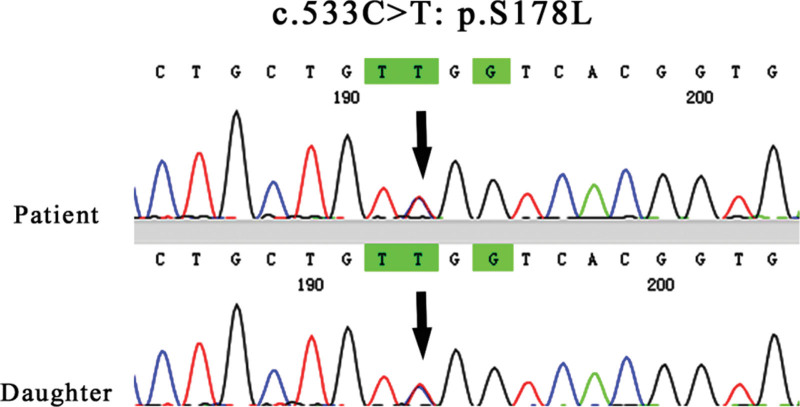
M1 mutation: SLC12A3:NM_000339.3:exon4:c.533C > T:p.S178L.

**Figure 2.**
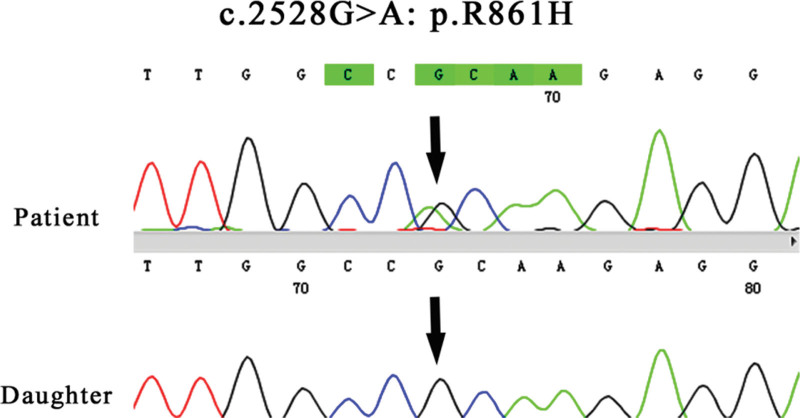
M2 mutation: SLC12A3:NM_000339.3:exon22:c.2582G > A:p.R861H.

Table 1 shows the laboratory data of the patient. During her hospital stay, the patient serum potassium and magnesium levels were 2.92 mmol/L and 0.5 mmol/L, respectively. Blood gas analysis showed that the PH was 7.47. The chloride excretion fraction (FE_Cl_) and magnesium excretion fraction were calculated as 3.4% and 9%, respectively. The urinary calcium/creatinine ratio was calculated as 0.24 mmol/mmol, and the value was 0.04 mmol/mmol on the next day reexamination. Both times, the RAAS was active. The cortisol rhythm and adrenocorticotropic hormone were normal. A HCT test was performed on the patient (Table 2), and the change value of the chloride excretion fraction was 0.43%. After obtaining the consent of the patient and her family, we collected peripheral blood from the patient and 1 of her daughters for whole-exome sequencing (including mitochondria). The results showed that the patient carried 2 heterozygous missense variants in the SLC12A3 gene, namely M1 variant: c.533C > T:p.S178L, a possible pathogenic variant; and M2 variation: c.2582G > A:p.R861H, which is the pathogenic variant. Unfortunately, due to her parents were not alive, it was not possible to determine the exact mechanism of inheriting the mutation. The patient daughter was as heterozygous as her M1, and no M2 variant was detected (Figs. 1 and 2). The 12-lead electrocardiogram (ECG) showed sinus rhythm, left axis deviation, low voltage in the chest lead, and a prolonged QTc interval. Abdominal ultrasound, adrenal CT, and pituitary MRI showed no abnormality.

**Table 1:** Laboratory examination of the patient.

Parameter	Test value	Reference range
Potassium (mmol/L)	2.92	3.5–5.5
Sodium (mmol/L)	138	137–147
Chlorine (mmol/L)	97.3	99–110
Calcium (mmol/L)	2.29	2.11–2.52
Phosphorus (mmol/L)	1.4	0.85–1.51
Magnesium (mmol/L)	0.5	0.75–1.02
Urinary potassium (mmol/24h)	80.42	25–125
Urinary sodium (mmol/24h)	242	40–220
Urinary chlorine (mmol/24h)	308	110–250
Urinary calcium (mmol/24h)	1.5	2.5–7.5
Urinary phosphorus (mmol/24h)	14.59	22–48
Urinary magnesium (mmol/24h)	4.34	
FE_Cl_ (%)	3.4	>0.5※
FE_Mg_ (%)	9	>4※
Urinary calcium/creatinine (mmol/mmol)	0.24	<0.2※
Arterial blood gas analysis		
Arterial blood PH	7.38	7.35–7.45
PCO_2_ (mm Hg)	47.2	35–45
BE (mmol/L)	2.7	−3 to 3
HCO3^−^ (mmol/L)	27.4	21–26
Recumbent RAAS		
Renin (pg/mL)	99.32	4–24
Angiotensin (pg/mL)	97.02	25–129
Aldosterone (pg/mL)	157.33	10–160
ARR	1.58	<40
Orthostatic RAAS		
Renin (pg/mL)	600.43	4–24
Angiotensin (pg/mL)	101.26	25–129
Aldosterone (pg/mL)	345.33	10–160
RR	0.58	<40
FPG (mmol/L)	7.26	3.89–6.11
HbA1c (%)	6	4.8–5.9
ΔFE_Cl_ (%)	0.43	≤2.86※

ARR = aldosterone renin ratio, BE = base excess, FE_Cl_ = fractional chloride excretion, FE_Mg_ = fractional magnesium excretion, FPG = fasting plasma glucose, HbA1c = hemoglobin A1c, HCO3^-^ = bicarbonate, PCO2 = partial pressure of carbon dioxide, RAAS = renin-angiotensin-aldosterone system, ΔFECl = change in fraction of chloride excretion.

※ : The threshold for diagnosis of GS.

**Table 2:** Data of the HCT test*.

Time (min)	FE_Cl_ (%)	FE_Clb_ (%)	FE_ClMax_ (%)	ΔFE_Cl_ (%)
30	2.56	2.54		0.43
60	2.51			
90	2.87		2.97	
120	2.97			
150	2.69			
180	2.92			
210	2.67			
240	2.48			

FE_Clb_ = basal clearances, mean of chloride clearance over the first two 30-min, FE_ClMax_ = maximal clearances for the last six 30-min, ΔFE_Cl_ = maximal increase.

* HCT test: the patients stopped supplementing potassium and magnesium for 1 d before the test, and the serum potassium level before the test was not lower than 3 mmol/L. Spontaneous urination was facilitated by drinking water (10 mL/kg) half an hour before the test. After two 30-min basal removals, the patient was given 50 mg of hydrochlorothiazide tablets orally, followed by 6 additional 30-min removals. At 60, 120, and 180 min, the patients were given 150ml water, respectively. Serum creatinine and chloride levels were measured at 60 min and 240 min. Urine was collected every 30 min. The value at which the maximum change in fractional chloride excretion exceeded the change in fractional chloride excretion at the basal level was selected as the final test result.

According to the typical symptoms, laboratory tests, and genetic testing, the patient was diagnosed with GS and diabetes. The clinical manifestation grade of GS classification was grade C, the hypokalemia grade was grade 3, and the hypomagnesemia grade was grade 2.

She was treated with potassium chloride sustained-release tablets, potassium magnesium aspartate tablets, spironolactone, and occasionally temporary oral administration of oral potassium chloride solution. Dapagliflozin was used to control the blood glucose.

**Figure 3.**
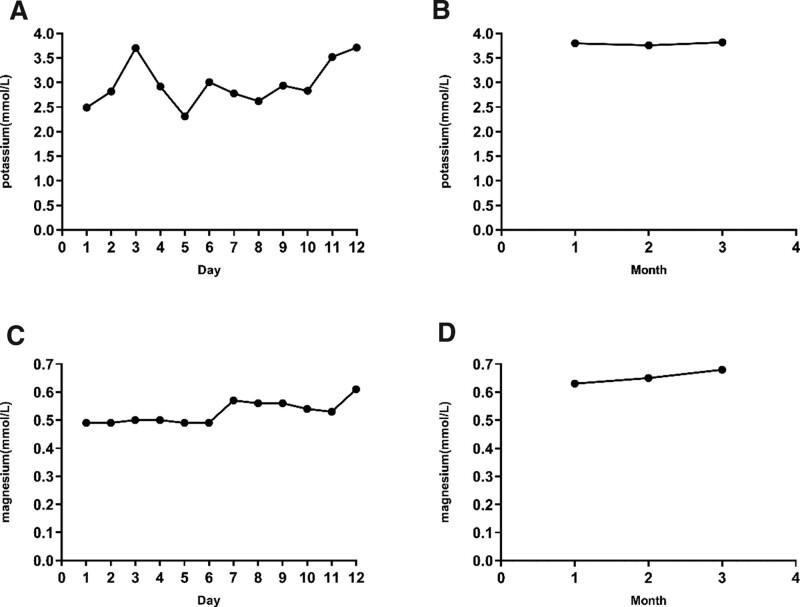
The patient serum electrolyte levels during hospitalization and 3-mo follow-up. A and C are the levels of serum potassium and magnesium of patients during hospitalization; B and D are the levels of serum potassium and magnesium within 3 mo of follow-up after discharge.

The patient took oral drugs regularly outside the hospital, and the symptoms of dizziness and fatigue were alleviated. During the 3-month follow-up period, the patient blood potassium level was normal, blood magnesium level was above 0.6 mmol/L (Fig. 3), and blood glucose was controlled smoothly.

## 3. Discussion

GS was first reported by Gitelman, Graham, and Welt in 1966.^[8]^ It is a rare genetic disease with a prevalence of 1 to 10/40000 in Western countries, and the prevalence is higher in the Asian population.^[1]^ The diagnosis of GS is mainly based on symptoms, laboratory tests, and genetic testing. The main laboratory findings were hypokalemia, hypomagnesia, hypocalcemia, metabolic alkalosis, and increased RAAS activity. Most of the symptoms were systemic manifestations of hypokalemia and hypomagnesia, such as salt intolerance, dizziness, fatigue, thirst, polydipsia, muscle weakness, hand-foot convulsions, normal or low blood pressure, and even cartilage calcification, arthralgia, and nocturia. This patient is consistent with the symptoms and laboratory findings of GS. However, given the clinical similarities between GS and Batter syndrome, we performed HCT trial, which is one of the chloride clearance tests. HCT is a commonly used antihypertensive drug and diuretic that can inhibit the reabsorption of sodium and chloride ions by inhibiting NCCT. The trial can identify the injured sites in renal tubules and reflect the functional status of NCCT. Colussi et al^[9]^ first conducted the chloride clearance test in 1997. Then they reconducted the HCT test in patients with GS confirmed by genetic testing in 2015,^[10]^ and it was found that 92.7% of the patients with GS had no obvious response, while patients with Batter syndrome and sham Batter syndrome had an obvious response. In the same year, Chinese scholar Peng Xiaoyan et al^[11]^ applied the improved chloride clearance test method to the Chinese population for the first time, and determined the cutoff value of the HCT test for the diagnosis of GS (ΔFE_Cl_ ≤ 2.86). The sensitivity, specificity, and coincidence rate of this method for the diagnosis of GS were 95.7%, 95.8%, and 95.7%, respectively. There was good agreement with gene diagnosis (Kappa = 0.915, *P* < .001). The ΔFE_Cl_ of this patient was 0.43, which was consistent with the diagnosis of GS. During the trial, the vital signs of the patients were stable, and no adverse events such as hypotension and hypokalemia, which have been mentioned in the consensus and guidelines of the KDIGO Controversies Conference, occurred.

More than 500 SLC12A3 mutations have been identified in GS patients. In the Chinese population, the most common mutations were p.T60M and p.D486N, whereas the most common mutations in the European population were p.A313V, c.1180 + 1G > T, p.G741R, p.L859p, p.R861c, c.2883 + 1G > T, and p.C994Y.^[12]^ In addition, some GS patients are caused by CLCNKB gene mutations,^[13]^ but the specific mechanism is still unclear. Two heterozygous missense variants in the SLC12A3 gene were identified in this patient. One was c.533C > T:p.S178L, which resulted in a C to T substitution at the 533 position of the cDNA, resulting in a serine to leucine substitution at the 178 position, which was a possible pathogenic variant. The other was c.2582G > A:p.R861H, where the 2582 position of cDNA was replaced by G to A, resulting in conversion of arginase to histidine at the 861 position, which was a pathogenic variation. But the source of the variation is unknown because the patient parents have died for more than 10 years. In the genetic testing, we found that her asymptomatic daughter also had c.533C > T:p.S178L.

Existing reports have found that GS patients have an increased risk of T2DM^[14,15]^ and an earlier age of onset.^[16,17]^ In Chinese GS patients, 14 to 60% may have abnormal glucose metabolism,^[18]^ which may be due to hypokalemia, hypomagnesemia, and increased RAAS activity. After the body ingests food, blood glucose concentrations rise and enter the cell via the insulin-dependent glucose transporter 2. Glucose is rapidly phosphorylated by glucose kinase to produce glucose-6-phosphate, and to produce an energy-rich ATP through further metabolism. The change in the ratio of ATP to ADP closes the ATP-sensitive potassium channels, leading to the accumulation of some intracellular potassium, which subsequently depolarizes the cell membrane, opening voltage-regulated calcium channels and triggering insulin secretion. Hypokalemia prevents the closure of ATP-sensitive potassium channels on pancreatic islet β cells, causing reduced insulin secretion and, in turn, causing disturbed glucose metabolism with further progression to T2DM.^[2]^ Magnesium is a key cofactor for several enzymatic reactions in glucose metabolism, involved in regulating the activity of all enzymes in phosphorylation. Magnesium deficiency can lead to a disorder of insulin receptor tyrosine kinase activity, leading to the occurrence of postreceptor insulin resistance and reducing cellular glucose utilization. Magnesium deficiency can also affect the glucose kinase activity, making the aforementioned ATP-sensitive potassium and calcium channels unregulated and then reducing insulin secretion. In addition, magnesium is also an anti-inflammatory factor, and hypomagnesemia increases the inflammatory environment in obese patients, significantly increasing the levels of IL-1 and TNF-α. Hypomagnesemia also leads to neutrophil activation along with oxidative stress. So hypomagnesemia can cause insulin resistance by increasing inflammation and oxidative stress.^[3,4]^ After the activation of RAAS, excessive aldosterone will increase the generation of reactive oxygen species and promote the remodeling of vascular endothelial cells,^[5]^ thereby reducing the delivery of insulin required for glucose metabolism. High aldosterone can also cause recombinant glucose transporter 4 gene expression deficiency by reducing insulin receptors, affecting the expression and phosphorylation of insulin receptor substrate-1 and protein kinase B, thereby reducing glucose uptake of skeletal muscle and adipocytes.^[6,7]^ In addition, excessive aldosterone can also reduce the expression of insulin receptor substrate-1 in vascular smooth muscle and block the downstream protein kinase B signaling pathway, leading to insulin resistance.^[19,20]^ However, some current studies show that glucose metabolism and insulin secretion are abnormal, but insulin sensitivity has not changed in GS patients.^[15]^ In Table 3, We summarized the general characteristics of GS patients with T2DM in published articles.^[21–26]^ Based on the results, the mutation sites in GS patients with T2DM are mostly located in the exon areas, and it is more common in Chinese male patients. Some patients were diagnosed with T2DM earlier than GS, so it is not clear whether the cause of T2DM in these patients is caused by GS. However, the results are limited due to fewer studies on GS patients with T2DM. Therefore, large prospective studies on the relationship between GS and T2DM are needed. At present, a variety of hypoglycemic drugs can be used to control blood glucose in GS patients with diabetes. A meta-analysis of 18 randomized controlled trials (including 15,309 patients with 4 types of Sodium-glucose cotransporter 2 inhibitors [SGLT2i]) published in 2016 showed that serum magnesium levels were significantly increased by using SGLT2i in patients without kidney disease (weighted mean difference: Canagliflozin 0.06 mmol/L [100 mg], 0.09 mmol/L [300 mg]; Dapagliflozin 0.1 mmol/L [10 mg]; Empagliflozin 0.04 mmol/L [10 mg], 0.07 mmol/L [25 mg]; Ipragliflozin 0.05 mmol/L [50 mg]).^[27]^ Therefore, SGLT2i can be chosen for GS patients with diabetes in order to increase serum magnesium level while controlling blood glucose. However, Ahmed et al^[28]^ reported a case of euglycemic Diabetic Ketoacidosis in a GS patient after using SGLT2i. Therefore, the use of SGLT2i should pay attention to the changes in blood volume. This patient had been diagnosed with diabetes for 7 years and was considered to be related to GS. We cannot distinguish whether the hyperglycemia of this patient is a result of type 2 diabetes or GS up to date. Dapagliflozin was administered to control blood glucose.

**Table 3:** The characteristics of patients with Gitelman syndrome coexisted with type 2 diabetes.

SLC12A3 gene	Gene mutation	Sex	Country	Age at onset of hypokaemia (yr)	Age of GS diagnosed (yr)	Age of T2DM diagnosed (yr)	Antidiabetic drug	Reference
Exon 1 Exon 25	158C > T -	M	Chinese Taiwanese	-	13	38	-	^[21]^
Exon 1 Exon 25	159C > T -	M	Chinese Taiwanese	-	21	42	-	^[21]^
Intron 13	IVS13-191C→T	M	Chinese Taiwanese	-	18	45	-	^[21]^
Intron 13	IVS13-191C→T	F	Chinese Taiwanese	-	18	40	-	^[21]^
Exon 1 Exon 3	158 C > T 513 C > T	M	Chinese Taiwanese	-	16	43	-	^[21]^
Exon 12 Exon 21	c.1567G > A C.2542G > A	F	Chinese	42	62	52	Dapagliflozin, alogliptin	^[22]^
Exon 8 Exon 6	c.1077C > G c.784_785insTCATTGGCGTGGTCTCGG	M	Chinese	38	55	55	Acarbose	^[23]^
-	c.2038-1delG c.2012 T > G	M	Chinese	21	40	40	Acarbose	^[24]^
-	c.179C > T c.1740delC	F	Chinese	54	55	51	Trajenta	^[25]^
-	c.137del c.2927C > T	M	Japanese	36	49	43	Glimepiride, insulin degludec	^[26]^
exon4 exon22	c.533C > T c.2582G > A	F	Chinese	41	51	44	Dapagliflozin	The present study

F = female, M = male, T2DM = type 2 diabetic mellitus.

One limitation of this case is that both of the patient parents were deceased, so there was no way to determine the source of the patient mutated gene. However, in order to better understand the genetic law of the mutated gene, the patient daughter underwent genetic testing. Another disadvantage is the insufficient follow-up time. Longer follow-up is required to monitor electrolyte and blood glucose changes in patients in order to better understand the relationship between hypokalemia, hypomagnesemia, and glucose metabolism. One advantage of this case is that we conducted HCT testing and genetic testing on the patient at the same time, which confirmed the damage of NCCT in the patient from the perspective of gene and function and finally confirmed the diagnosis. Another advantage is that we elaborated on the relevant mechanisms of GS and glucose metabolism disorders.

## 4. Conclusion

When GS is considered in patients with unexplained hypokalemia, the HCT test can be used for differential diagnosis, and genetic testing can be continued to confirm the diagnosis when conditions are available. GS patients often have abnormal glucose metabolism, which is mainly caused by hypokalemia, hypomagnesemia, and secondary activation of RAAS. When a patient is diagnosed with GS and type 2 diabetes, SGLT2i can be used to control the blood glucose level and assist in raising blood magnesium.

## Author contributions

**Conceptualization:** Hao Qi, Jing Yang.

**Supervision:** Hao Qi, Jing Yang.

**Writing – original draft:** Luyang Yang, Jinmeng Fan.

**Writing – review & editing:** Luyang Yang, Jinmeng Fan, Yunfeng Liu, Yi Ren, Zekun Liu, Hairui Fu.
